# Polycyclic aromatic hydrocarbons and PAH-related DNA adducts

**DOI:** 10.1007/s13353-016-0380-3

**Published:** 2016-12-12

**Authors:** Błaszczyk Ewa, Mielżyńska-Švach Danuta

**Affiliations:** 1 Institute for Ecology of Industrial Areas, Environmental Toxicology Group, 6, Kossutha Street, 40-844 Katowice, Poland; 2 Witold Pilecki State School of Higher Education, 8, Maksymiliana Kolbego Street, 32-600 Oświęcim, Poland

**Keywords:** Polycyclic aromatic hydrocarbons, Benzo[a]pyrene, DNA damage, PAH–DNA adducts

## Abstract

Investigations on the impact of chemicals on the environment and human health have led to the development of an exposome concept. The exposome refers to the totality of exposures received by a person during life, including exposures to life-style factors, from the prenatal period to death. The exposure to genotoxic chemicals and their reactive metabolites can induce chemical modifications of DNA, such as, for example, DNA adducts, which have been extensively studied and which play a key role in chemically induced carcinogenesis. Development of different methods for the identification of DNA adducts has led to adopting DNA adductomic approaches. The ability to simultaneously detect multiple PAH-derived DNA adducts may allow for the improved assessment of exposure, and offer a mechanistic insight into the carcinogenic process following exposure to PAH mixtures. The major advantage of measuring chemical-specific DNA adducts is the assessment of a biologically effective dose. This review provides information about the occurrence of the polycyclic aromatic hydrocarbons (PAHs) and their influence on human exposure and biological effects, including PAH-derived DNA adduct formation and repair processes. Selected methods used for determination of DNA adducts have been presented.

## Introduction

It is well known that exposure to toxic chemicals can cause many harmful health effects, among which the most important, both for the individual and the whole population, are cancer and genetic defects in the offspring of the exposed populations. In research papers, the estimated range of cancer cases caused by environmental exposure varies from 1 to 100%. Differences in the above range are mainly associated with variations in the definitions of “environmental” factors (Parker 2014). A broader approach to the term “environmental” indicates that around 90–95% of human cancers result from exposure to exogenous and endogenous agents, including lifestyle and health behavior such as tobacco smoking, diet, infections, sun radiation, stress, obesity, physical activity, as well as environmental pollutants from air, water and soil, etc. It is also estimated that genetic factors are responsible for 5 to 10% of these cases (Anand et al. 2008). World Health Organization reports that 19% of all cancers are globally attributable to environmental factors, but it refers to a limited number of factors, i.e., air, water and soil chemical pollutants, or biological agents, including occupational exposures (Prüss-Üstün and Corvalan 2006). Most substances are classified as non-threshold carcinogenic substances, which means that no safe levels of exposure can be determined for them. Carcinogenic compounds do not differ in their properties from other xenobiotics. Most of them in some ranges demonstrate a dose–response effect, undergo transformation and degradation in the environment through chemical and biological processes and react with other xenobiotics. Chemical compounds can enter the human body through different pathways, and then they can be metabolized, accumulated, and transported to organs, which consequently may result in permanent damage and even diseases (Esteban and Castano 2009; Manzetti 2013).

Exposure to genotoxic factors occurs not only in the workplace, but it is also connected with pollution of the natural environment (air, water, soil), therapeutic procedures (radiotherapy, chemotherapy) and lifestyle, i.e., diet, smoking, alcohol consumption, taking medicines, and use of cosmetics and detergents, as well as sexual behaviors (Wogan et al. 2004). Factors related to unhealthy lifestyle are one of the main risk factors of cancer associated with environmental exposure (Weiderpass 2010).

**Fig. 1:**
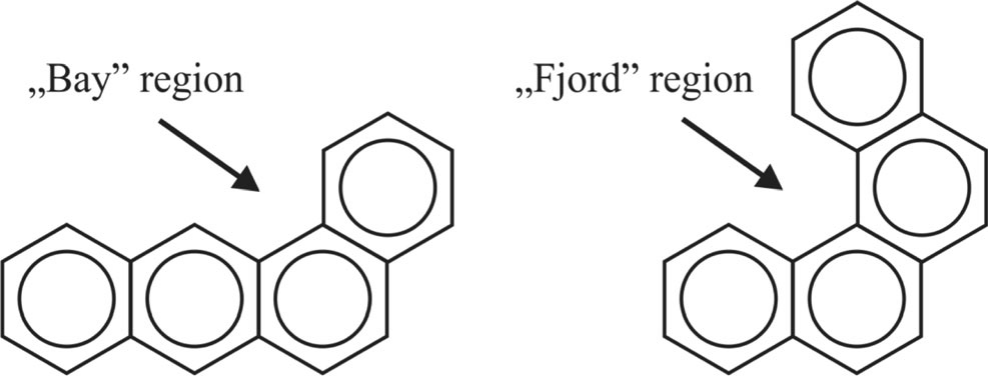
“Bay” and “fjord” regions in different PAH conformations

Adduct formation is the result of a covalent binding between reactive electrophilic substances and the nucleophilic sites in DNA and proteins. The ability of a chemical to bind to DNA, either directly or after metabolic activation, is taken as an evidence of mutagenic and carcinogenic potential. The group of compounds with well-established genotoxicity are polycyclic aromatic hydrocarbons (PAHs). The biological activity of these compounds is connected with their structural features, formed between angular condensed aromatic rings possibly as a result of distortions in a region with maximal impact, termed as “fjord” or “bay” regions (Fig. 1). It is obvious that reactivity depends directly on density of an electron charge. However, geometric distortions in molecules influences charge distribution and indirectly also its reactivity in certain positions. Molecules with “fjord” regions (e.g., dibenzo[a,l]pyrene) are generally non-planar and bind preferentially to adenine nucleotides. On the other hand, PAHs with a “bay” region (e.g., B[a]P) are planar and bind to guanine nucleotides. Furthermore, increasing the non-planarity of PAHs lowers their capability of being metabolized to reactive forms which produce DNA-damaging adducts (Lakshman et al. 2000; Muñoz and Albores 2011).

It has already been shown that DNA adducts are involved at early stages of carcinogenesis. DNA adduct formation is necessary but not sufficient for tumor induction, and there are many additional factors which contribute to carcinogenesis (Poirier 2016). PAH–DNA adducts are measured extensively in biomonitoring studies to examine exposure to environmental, dietary, lifestyle, and occupational chemicals, etc. It was observed that genotoxic effects were dose-dependent, and the DNA adduct level increased with the increased B[a]P concentration (Whyatt et al. 1998; Sinha et al. 2005; Singh et al. 2007; Pavanello et al. 2008; McCarty et al. 2009). Verma et al. (2012). Furthermore, many studies show that the presence of PAH–DNA adducts in blood or other organ cells is associated with an increased relative risk (1.3–7.7) of different types of cancer (Tang et al. 1995, 2013; Chen et al. 2002; Zhu et al. 2003; Gammon et al. 2004; Gunter et al. 2007).

### Polycyclic aromatic hydrocarbons — occurrence and human exposure

Polycyclic aromatic hydrocarbons (PAHs) are a large group of organic compounds with two or more fused aromatic rings. They have a relatively low solubility in water, but are highly lipophilic. In addition, aqueous solubility decreases for each additional ring added to PAHs (Srogi 2007). The behavior of PAHs in the atmosphere depends on complex physico-chemical reactions, interactions with other pollutants, and photochemical transformations, as well as dry and wet deposition. Ubiquitous occurrence and environmental processes which PAHs are undergoing contribute to their impact on humans, flora, fauna, water, air, and soil (Kim et al. 2013). Carcinogenic, mutagenic, and cytotoxic properties have been confirmed for polycyclic aromatic hydrocarbons. Numerous epidemiological and toxicological studies confirm a strong correlation between exposure to PAHs and an increased risk of cancer incidence. PAHs are formed in the pyrolysis and incomplete combustion of organic matter of both natural and anthropogenic origin. These pollutants do not occur in the environment in the form of single compounds—they are always composed of a multicomponent mixture. Qualitative and quantitative composition of these mixtures depends on the type of burned material and the conditions under which the combustion process takes place (Sapota 2002).

Natural sources include forest and meadow fires, volcanic eruptions, reactions of humus compounds under the influence of soil microorganisms leading to the formation of coal or oil, and biosynthesis carried out by bacteria, algae, and plants. It should be noted here that the share of natural resources in the emission of PAHs into the environment is marginal. The major sources of these substances are industrial processes associated with burning of oil and coal (coke, aluminum production, or processing of coal tar), and burning in the municipal sector, as well as exhaust gases from various types of engines, especially diesel and tobacco smoke (Klimaszewska 1999; Mielżyńska 2008). Tobacco smoke contains high concentrations of PAHs. PAHs occur in various environmental compartments, such as air, water, soil, sediments, and thermally treated food (frying, baking, grilling, smoking), as well as in pharmaceutical products based on coal tar that are applied to the skin (IARC 2010). Average concentrations of individual PAHs in the ambient air in urban areas generally range from 1 to 30 ng/m^3^. However, concentrations up to several tens of nanograms per cubic meter have been reported in road tunnels or in large cities, where extensive use of coal or other biomass as residential heating fuel has been recorded. Estimates of PAH intake from food vary widely, ranging from a few nanograms to a few micrograms per person daily (IARC 2012).

Occupational exposure to PAHs occurs primarily through inhalation and via skin contact. Exposure to benzo[a]pyrene was measured in such industries as the following: coal liquefaction, coal gasification, coke production and coke ovens, coal-tar distillation, roofing and paving (involving coal-tar pitch), wood impregnation/preservation with creosote, aluminum production (including anode manufacture), carbon-electrode manufacture, chimney sweeping, and power plants. The highest levels of exposure to PAHs can be observed in aluminum production, with values up to 100 μg/m^3^. Mid-range levels are recorded in roofing and paving, whereas the lowest concentrations are observed in coal liquefaction, coal-tar distillation, wood impregnation, chimney sweeping and in power plants (IARC 2010, 2012).

The best known PAH compound is benzo[a]pyrene (B[a]P), which in 2012 was classified among the highly genotoxic compounds. According to the International Agency for Research on Cancer (IARC) it belongs to group 1—carcinogenic to humans (IARC 2012). Moreover, the products containing B[a]P and other PAHs (tobacco smoke, indoor emissions from household combustion of coal, diesel exhaust fumes, outdoor air pollution, and particulate matter) are also classified to group 1 (IARC 2016). Numerous animal studies confirm carcinogenic properties of B[a]P. Exposure to B[a]P and/or its mixture causes immunotoxic, teratogenic effects, and induces apoptosis and cell proliferation, as well as increased DNA methylation. In population studies concerning exposure to the mixture containing this compound, a relationship between exposure and the development of cancers has been proven. In humans, occupational exposure to benzo[a]pyrene-containing mixtures was associated with different kinds of cancer: (1) coke production — lung cancer, (2) coal gasification — lung and bladder, (3) paving and roofing — lung, (4)coal-tar distillation — skin, (5) soot — lung, oesophagus, haematolymphatic system and skin, (6) aluminum smelting — lung and bladder, and (7) tobacco smoking — lung, lip, oral cavity, pharynx, oesophagus, larynx, and bladder (IARC 2010, 2012).

### PAH–DNA adducts — from biotransformation to DNA damage formation and repair processes

**Fig. 2:**
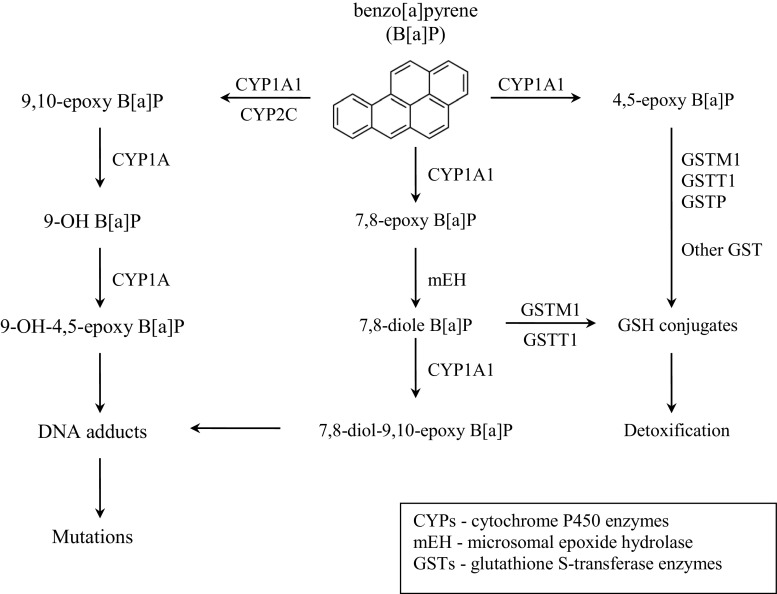
Metabolic activation pathways of benzo[a]pyrene (Lodovici et al. 2004)

Xenobiotic metabolism usually occurs in the liver. Most xenobiotics, like PAHs, are lipophilic, bind to lipid membranes and are transported by lipoproteins in the blood. After entrance to the body via lungs, digestion tract, and/or skin, PAHs may undergo one or two phases of metabolism. In phase I, a polar reactive group is introduced into the molecule, rendering it a suitable substrate for phase II enzymes. Phase I reactions include microsomal monooxygenations, cytosolic and mitochondrial oxidations, co-oxidations in the prostaglandin synthetase reactions, reductions, hydrolyses, and epoxide hydration. In phase II, following the introduction of a polar group, conjugating enzymes usually add endogenous substituents, such as sugars, sulfates, or amino acids, which substantially increases water solubility, making it easily excreted. Although this process is generally a detoxication sequence, reactive intermediates may be much more toxic than the parent compounds (Guengerich 2008; Hodgson and Rose 2010). In phase I, three main pathways of PAH activation can be distinguished: (i) formation of dihydrodiol epoxides catalyzed by cytochrome P450 enzymes and epoxide hydrolase (CYP/EH pathway), (ii) formation of a PAH radical cation in a metabolic oxidation process by cytochrome P450 peroxidase activity, and (iii) formation of ortho-quinones via oxidation of catechols by dihydrodiol dehydrogenase, a member of aldo–keto reductase (AKR pathway) (Guengerich 2008; Shimada 2006). Redox cycling of quinones could lead to formation of ROS, which could also lead to carcinogenesis via oxidative DNA damage (Moorthy et al. 2015). While the epoxide pathway leads to the formation of stable DNA adducts, the radical cations generate labile DNA adducts that are eliminated via depurination, resulting in apurinic sites (Henkler et al. 2012). The most common mechanisms of metabolic activation of PAHs, such as B[a]P, are involving and generating a large number of metabolites due to the activity of phase I (activation) and phase II (detoxification) enzymes. In the phase I oxidation, reactions catalyzed by cytochrome P450 enzymes (CYPs: 1A1, 1A2, 1B1, 3A4) and hydroxylation by epoxide hydrolase occur. CYP1A1 or CYP1B1 are highly inducible by the exposure to PAHs via the aryl hydrocarbon receptor (AhR). The AhR is present in the cytoplasm as a complex with other proteins such as heat shock protein 90 (Hsp90), p23, and AhR-interacting protein. Having formed a complex with PAHs, the Hsp90 is released and an AhR–PAH complex is translocated to the nucleus. There, the AhR–PAH complex creates a heterodimer with an ARNT (AhR nuclear translocator), and afterwards binds to DNA via the xenobiotic response element (XRE) situated in the promoter region of CYP1A and CYP1B genes. Therefore, the AhR plays an important role in the tumorigenesis mediated by PAHs, which has been illustrated previously (Shimada 2006; Arenas-Huertero et al. 2011). The obtained diol epoxides are hydrophilic and can dissolve in water more easily. For this reason, they are involved in the phase II reactions, i.e., coupled with endogenous compounds — sulfuric, glucuronic acid, or glutathione (Mielżyńska 2008; Moździerz et al. 2010). Some of the PAH metabolic intermediates show genotoxic and carcinogenic properties (Xue and Warshawsky 2005; IARC 2012). Studies examining the carcinogenicity of B[a]P have identified the 7,8-oxide B[a]P and 7,8-dihydrodiol B[a]P as proximate carcinogens, and the 7,8-diol-9,10-epoxide B[a]P (BPDE) as a strong mutagen and ultimate carcinogen (Fig. 2).

One of the well-known PAH compounds is B[a]P, recognized as an indirect mutagen (procarcinogen), which after metabolic transformation to active derivatives of electrophilic properties can form covalent bonds with DNA (ATSDR 1995). DNA adducts are compounds which carcinogenic substances form with cellular macromolecules. Since most of the carcinogenic compounds or their metabolites are electrophiles, they can covalently bind with nucleophilic sites in proteins or DNA (Phillips 2005). Protein adduct formation is considered to be an alternative of DNA adduct formation, but only the latter results in critical mutagenic changes. The level of specific DNA adducts is commonly considered to be a biomarker of the biologically effective dose, and if the adducts are able to induce mutations leading to cancer, they may also be recognized as biomarker of effect (Henderson 2005). DNA adduct formation during the extent of reaction with a particular nucleic acid base will depend on the particular stereoisomer. The site of substitution for biologically important adducts appear to be chemical-class specific (Beland and Poirier 1994). For PAHs, one of the activation pathways is the formation of dihydrodiol epoxides that covalently bind to exocyclic amino groups of purines in DNA to form stable adducts. Another pathway involves the formation of radical cations that bind to the N7 or C8 of purines to form unstable adducts and generate apurinic sites in DNA by spontaneous depurination (Melendez-Colon et al. 1999). The preferred sites for PAH–DNA adducts formation are the amino group of guanine with 5-nucleophilic sites, and to a lesser extent can bind to the adenine and cytosine (Beland and Poirier 1994). In the case of B[a]P metabolites, DNA adducts preferentially react with N2 of guanine (i.e., dG-N^2^–BPDE) and/or N6 of adenine (i.e., dA-N^6^–BPDE). Reactions with DNA are clearly non-random. What is more, higher-order chromatin structure affects the binding of carcinogens to DNA (Beland and Poirier 1994). Adduct generation is a pre-mutation change, and in most cases it is recognized and processed by repair systems. However, adducts that have not been removed can initiate a point mutation in the form of substitution or deletion, which is considered to be the first step in the development of cancer. Formation of stable PAH–DNA adducts can lead to the induction of mutations that activate proto-oncogenes or inactivate tumor suppressor genes as an important event during tumor initiation (Melendez-Colon et al. 1999).

PAH exposures, in addition to causing DNA adduct formation, also induce oxidative stress that provokes mutation. If DNA repair mechanisms work insufficiently, the result is the accumulation of mutations in DNA, which may induce carcinogenesis. Activation of several molecular and cellular responses is associated with genes involved in apoptosis, cell-cycle control and DNA repair (Castorena-Torres et al. 2008). DNA damage responses utilize distinct checkpoints to delay cell cycle progression, in order to provide an opportunity for the repair of lesions. Depending on the level of damage, this cascade can either delay a cell-destructive response or trigger activation of programmed cell death (Hoeijmakers 2001). Molecular studies have revealed that DNA adducts block polymerase replication activity, contributing to the increased DNA damage by reducing the repair activity (Hsu et al. 2005). For B[a]P and other PAHs, the magnitude of DNA adduct formation depends on the metabolic capacity of the target cells (Boysen and Hecht 2003). However, a recent study carried out by Henkler et al. (2012) has demonstrated a sufficient metabolic capacity to generate mutagenic metabolites of B[a]P and to trigger a significant formation of PAH–DNA adducts even in human skin. It has been reported that B[a]P derivatives have the capacity to enter redox cycles and induce the production of reactive oxygen species (ROS), thereby causing oxidative stress (An et al. 2011). Free radicals generated this way react with guanine and cause DNA damage, including the production of 8-Oxo-2′-deoxyguanosine (8-oxo-dG). Oxidative DNA damage, such as 8-oxo-dG, may contribute to carcinogenesis by the mechanism involved in modulation of gene expression and through the induction of mutations (Valavanidis et al. 2013). Another pathway is activation of estrogen receptors (ER) and metabolism by steroid hormones. Compounds such as PAHs have the ability to displace natural estrogens and occupy ER binding sites (Vondráček et al. 2002; Plíšková et al. 2005). Nevertheless, the most important mechanism is a deficient DNA repair system responsible for removing cumulative mutations from key genes involved in cell cycle control, which leads to carcinogenesis. Differences in response to PAH exposure and individual human susceptibility is affected by genetic polymorphisms in many genes regulating enzymes involved in activation, detoxification, and repair processes (Pavanello and Lotti 2014). The most common mechanisms of repair activated after exposure to PAHs are: nucleotide excursion repair (NER), base excision repair (BER), recombinant repair and transcription-coupled repair (TCR). NER is the most important mechanism for the removal of bulky DNA adducts caused by PAHs (Braithwaite et al. 1998). In the case of this mechanism, two subpathways can be distinguished: GG-NER (global genomic-NER) and TC-NER (transcription-coupled-NER). The first corrects damage in transcriptionally silent areas of the genome, while the second one repairs lesions on the actively transcribed strand of DNA. Nucleotides that are lesioned due to depurination, deamination, alkylation, or ROS-mediated oxidation can be eliminated via BER mechanism (Sancar et al. 2004). The role of other mechanisms is still not clear, but homologous recombination (HR) is related to some extent to the repair of PAH–DNA damages. Also, mismatch repair (MMR) probably takes part in elimination of oxidative DNA damages caused by PAHs (Li et al. 2016). To a minor extent, chemical-induced DNA adducts are removed by MMR. Although it is evident that DNA adducts activate the DNA repair mechanisms, it is still not well recognized how these lesions trigger a cell-cycle arrest or apoptosis in the lesioned cells (Wu et al. 1999; Friedberg 2003).

### Methods for identification of DNA adducts

Analyses can be performed on various samples including tissues, isolated cells, and intact or hydrolyzed DNA from a variety of biological samples used in human monitoring. Sensitivity and specificity are considered to be the key factors for selecting the type of method for assessing the DNA damage. In particular, certain procedures can lead to the decrease of DNA adduct stability, which may have a significant impact on the determination of their amount. Several factors, such as sample preparation, storage conditions, handling methods, and so on can influence the outcome of the DNA adduct analysis. The amount of DNA needed for the analyses depends on the method and ranges widely, from <1 μg to 3 mg. Among the techniques which enable identification and quantification of adducts, the following techniques can be distinguished: techniques based on gas chromatography (GC) or high-performance liquid chromatography (HPLC) with electron capture detection (ECD), electrochemical or fluorescence detection (FD) with single (MS) or tandem (MS/MS) mass spectrometry or accelerator mass spectrometry (AMS). Other techniques are based on radioactive labeling (^14^C, ^3^H), ^32^P-postlabeling or histochemical and immunological methods. In the studies of adducts the PCR technique (i.e., ligation-mediated PCR) can also be applied, as well as non-specific methods, such as the comet assay. These methods differ mainly in sensitivity, and range from ∼1 adduct in 10^4^ to 10^12^ of nucleotides. All the above-mentioned techniques, except for immunohistochemistry, require DNA isolation, separation, and detection of adducts. On the other hand, however, in the analyses based on immunochemistry the use of antibodies is necessary. Furthermore, monoclonal antibodies are more specific than polyclonal antibodies, whereas polyclonal antibodies usually have greater sensitivity (Himmelstein et al. 2009). Each approach presents different advantages and limitations, and the most appropriate method depends on the type of the sample, level of damage, and nature of the investigation, as well as practical considerations (Brown 2012). In Table 1, a comparison of analytical methods used for quantification of DNA adducts is presented.

**Table 1:** Summary of analytical methods used for quantification of DNA adducts (Himmelstein et al. 2009)

Methods	Sensitivity	Amount of DNA required	Advantages	Limitations
Accelerator mass spectrometry (AMS)	∼1–10 adduct/10^12^ nt	1–1,000 μg	High sensitivity; adducts originate from radiolabeled compound	Various requirements including the need for ^14^C/^3^H-labeled compound, complex sample preparation, and good purification of DNA to prevent interferences with contaminants including protein, specialized equipment not widely available, limited specificity due to the lack of structural information
GC-ECD	∼1 adduct/10^11^ nt	100 μg	High sensitivity	Requires (laborious) derivatization, internal standards, and specialized equipment
^32^P-postlabeling	∼1 adduct/10^10^ nt	1–10 μg	Low amount of DNA required; sensitive and versatile	High levels of radioactivity are required; measures general damage but has limited specificity; adduct standards required for co-chromatography studies
HPLC-MS/MS	∼1 adduct/10^9^ nt	10–100 μg	Structural identification; high accuracy (MRM mode)	Requires specialized equipment; may require internal standards
GC-MS	∼1 adduct/10^9^ nt	10–500 μg	Structural identification	Requires derivatization, internal standards, and specialized equipment; in some GC-MS techniques there is a high risk of artifactual oxidative DNA damage
Radiolabeled binding assay	∼1–10 adduct/10^8^ nt	0.5–3 mg	Simple (if negative result); adducts originate from radiolabeled compound	Radiolabeled (^14^C or ^3^H) compound required; no structural information available; interference with contaminants including protein (requires good purification of DNA)
HPLC fluorescence or electrochemical detection	∼1–10 adduct/10^8^ nt	20–100 μg	Simple; robust method; inexpensive; the use of standards enables a limited amount of structural information	Only applicable for fluorescent/ electro-chemically active adducts; standards required
Immunoassay	∼2.5 adduct/10^8^ nt	1–200 μg	Applies antibodies targeted at a specific DNA adduct	Risk of cross-reactivity with other adducts
Immunohistochemistry	Variable	–	Robust and easy; allows localization of adducts	Poor identification; semi-quantitative; limited structural information available based on the applied antibody

### Methods for identification of PAH-related DNA adducts

In recent years, one of the methods frequently used in the studies of DNA adducts with aromatic compounds including PAHs was ^32^P-postlabeling technique (Szyfter et al. 1994; van Delf et al. 2001; Teixeira et al. 2002; Taioli et al. 2007; Umbuziero et al. 2008; Topinka et al. 2009; Wilson et al. 2011). The main advantage of this method is its high sensitivity and the low amount of DNA required, but it is characterized by limited specificity, because it detects stable adducts representative of the whole group of PAHs, called bulky DNA adducts (Himmelstein et al. 2009). What is more, nowadays due to the harmful effect of high radioactivity its application is limited.

Another group of methods based on immunological reactions used in various studies for detection of specific BPDE-1-DNA adducts includes: immunohistochemical techniques (IHC) (Santella and Zhang 2011), chemiluminescence immunoassay (CIA) (Divi et al. 2002; John et al. 2010), enzyme-linked immunosorbent assay (ELISA) (Schoket 1999; Whyatt et al. 2001; Topinka et al. 2009; Borska et al. 2014), or dissociation-enhanced lanthanide fluoroimmunoassay (DELFIA) (Divi et al. 2002). Identification of B[a]P-specific adducts such as diol epoxides of benzo[a]pyrene (BPDE) was developed with polyclonal and monoclonal antibodies to recognize DNA lesions. The first studies on BPDE–DNA adducts were validated in mouse cells and human white blood cells treated in vitro with B[a]P (Poirier et al. 1982; Santella et al. 1985; van Schooten et al. 1991). Immunochemical techniques were used in human lymphocytes occupationally and environmentally exposed to PAHs (Motykiewicz et al. 1995). BPDE–DNA adducts have also been determined in oral, urothelial, ovarian, and cervical cells, as well as blood vessels of smokers and non-smokers (Zhang et al. 1995, 1998; Hsu et al. 1997; Zenzes et al. 1998; Mancini et al. 1999; Romano et al. 1999). In epithelial cells from the oral cavity, these specific adducts were evaluated in environmentally exposed women from Silesia, Poland (Motykiewicz et al. 1998). In children blood lymphocyte BPDE–DNA adducts were identified using ELISA method (Borska et al. 2014).

An alternative to semi-quantitative immunochemical techniques is the analysis of DNA adducts using high-performance liquid chromatography with fluorescence detection (HPLC/FD), which was improved in further studies on human lung tissue (Alexandrov et al. 1992). In white blood cells, this method was successfully applied in populations occupationally exposed to PAHs and in tobacco smokers (Rojas et al. 1995; Pavanello et al. 1999, 2004; Mooney et al. 2005; Rundle et al. 2007). BPDE–DNA adducts were identified in the blood and cord blood of mother and newborn children from four different populations exposed to ambient PAHs (Perera et al. 2005).

## Conclusions

The identification of toxic chemicals that enter the body from exogenous sources, such as air pollutants, radiation, water contaminants, food, and drugs, must be carried out together with endogenous chemicals derived from cellular metabolism or endogenous processes, including inflammation, oxidative stress, infection, and chemicals derived from complex interactions with the intestinal flora (Balbo et al. 2014). The interaction of different PAHs may lead to additive, synergistic, or antagonistic effects in terms of DNA adduct formation and carcinogenic activity resulting from changes in metabolic activation of reactive intermediates and DNA repair (Singh et al. 2010). The investigation of these effects has led to the development of adductomic approaches to the investigation of protein adducts and DNA adducts. DNA adductomics is a relatively new field, and with recent improvements in sensitivity, liquid chromatography–mass spectrometry (LC–MS) is primed to replace 32P-postlabeling as the preferred approach to DNA adduct screening in humans because of its selectivity, specificity, and the structural information it provides. Unfortunately, some improvements in sample preparation and cleanup are still required, especially when detection of hydrophilic adducts is taken into account (Balbo et al. 2014). On the other hand, in the case of the previously mentioned immunohistochemical techniques, which are rarely discussed in scientific publications, evaluation, and validation of their sensitivity would be necessary.

Summing up, measurements of the DNA adducts may be carried out using various methodologies, but no single or selected battery of analyses can be recommended as the most useful for application to risk assessment because all these techniques have their own specific advantages, and this means that the choice of the biomarker should be made on a case-by-case basis (Himmelstein et al. 2009).
